# A 24-Week Treatment of Pediatric Hemangioma with Oral Propranolol

**Published:** 2017

**Authors:** Aziz Eghbali, Shabnam Hajiani, Bahman Sadeghi Sedeh, Abdolghader Pakniyat, Vahid Mansouri, Bahador Bagheri

**Affiliations:** a *Department of Pediatrics, Arak University of Medical Sciences, Arak, Iran.*; b *Department of Social Medicine, Arak University of Medical Sciences, Arak, Iran.*; c *Student Research Committee, Arak University of Medical Sciences, Arak, Iran.*; d *Proteomics Research Center, Shahid Beheshti University of Medical Sciences, Tehran, Iran.*; e *Cancer Research Center and Department of Pharmacology, Semnan University of Medical Sciences, Semnan, Iran.*

**Keywords:** Hemangioma, Propranolol, Children, Drug therapy, Hospital

## Abstract

Hemangioma is a benign vascular tumor that shouldbe treated in problematic situations.Propranolol efficacy, target dose, range of age, duration of treatment and complications arenot conclusive for treatment of pediatric hemangioma. Our goal was to study efficacy and safety of propranolol for hemangioma treating in children. A randomized, open label crossover trial with two twenty four-week treatment phases separated by a one-week washout period, was conducted in Amir-Kabir Hospital, Arak, Iran. Thirty two patients with age of 1 month to 15 years were randomized to receive either oral propranolol 2 mg/Kg/day or receivedno treatment. The primary outcome measure changed in hemangioma size assessed at baseline, day 3, day 7, and every month. At baseline, the mean surface area was 36.9 ± 36.3 cm^2^. After 1 week of treatment, a decrease was seen in size of hemangiomas. After one month, a significant reduction was seen in size of lesionsin treatment group compared to observation group (30 cm^2^vs 16 cm^2^, *P *< 0.01). Significant reductions were present at other intervals (*P *< 0.05). In the second phase of the study, a significant reduction was observed only after one month of treatment (*P *< 0.05). The trial suggested that 24 week treatment with oral propranolol was effective for treatment of pediatric hemangiomas with acceptable safety profile.

## Introduction

Hemangiomas are common benign vascular tumorsoccurring in 5% to 10% of neonates and approximately in 10% of children older than 1 year. Hemangiomais are morefrequently in females, particularly with low birth weight (1, 2). Hemangioma may be present at birth but it is generally recognized a few weeks after birth.The most common form of hemangioma is cutaneous;however organs like the brain, lungs, intestinal tract, liver, orbit, and airway may be involved.Hemangioma may lead to complications like ulceration, infection, hemorrhage, and scarring.More than 80% of cases will undergo spontaneous involution but some of them will become problematic and need medical interventions(3).According to the location and size of hemangioma, the appropriate treatment is chosen. Corticosteroids, interferon alfa, and vincristine have been extensively used for complicated and refractory hemangiomas, albeit these medical treatments are associated with variable responses and safety concerns exist about them(4-7). An old β-adrenergic antagonist, propranolol,hasrecently gained prominence in the treatment of infantilehemangioma (8). Propranolol-induced vasoconstriction immediately impacts hemangioma and causes size of hemangioma to decrease (9). At present, there is a paucity of data aboutinitiation and duration of treatment, range of age,target dose, and formulation of propranolol for treating pediatric hemangioma. This was the aim of our study. Here we report the efficacy of propranolol for pediatric hemangioma in a 6month treatment period.

## Methods

*Trial design*

The study was conducted as a randomized, open label, crossover trial in Amir Kabir Hospital, Arak, Iran. The local Ethics Committee approved the study and written informed consent was taken from parents. The study was conducted in accord with the European directive 2001/20/EC. The registration number of this trial corresponding to the Iranian Registry of Clinical Trials was IRCT2015011920715N1. Thirty seven children younger than 15 years old who had skin hemangiomawith surface area of bigger than 35 cm^2^were included in the study. Patients were randomly assigned to either propranolol or no treatment for 24 weeks followed by a 1-week washout period before they crossed over to the next phase which lasted 24 weeks. Patients were given oral propranolol 2 mg/Kg/day (Abidi, Tehran, Iran) divided into twice daily doses for 6 months. Propranolol was tapered in the last month of treatment. The exclusion criteria were diabetes, asthma, overt heart failure, hypersensitivity, severe sinus bradycardia, deep visceral hemangioma, pheochromocytoma, and cardiogenic shock. 

*Efficacy assessment*

The patients were evaluated at baseline, day 3, day 7 and then every month. Outcome for hemangioma improvement or worsening including size, color, swelling, redness and thickening were assessed and recorded by blinded pediatricians at each visit. 

*Safety assessment*

Ateach visit, physical examination, and assessment of vital signs were done by blinded pediatricians. Untoward effects of propranolol including hypotension, bradycardia, hypoglycemia, and bronchospasm were closely monitored. Parents were informed not to discontinue the drug without physician advice and they were trained about serious side effects of propranolol. 

*Data analysis*

The Data are shown in mean ± SD. The Analyses were done using *t* test and x^2^.*P *< 0.05 was considered as statistical significance. The Analyses were carried out using SPSS software version 19.0, Chicago, USA.

## Results

*Baseline characteristics*

**Figure 1:**
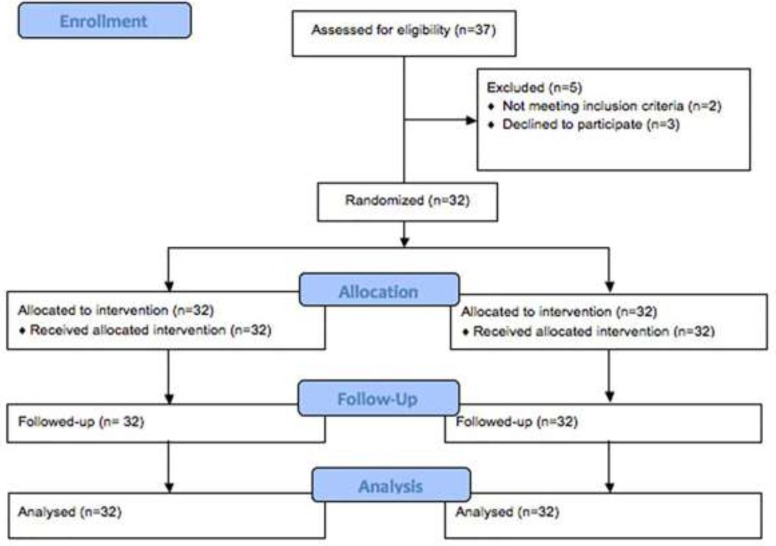
Consort diagram detailing the study subjects

Of the 37 patients who had been assessed for eligibility, 5 patients did not enter the randomized treatment. A total of 32 patients were studied between September 2014 and September 2015. Patients flow through the study is presented in Figure 1. Clinical characteristics of study subjects are shown in Table 1. The mean age was 39.6 ± 4.4 months with an excess of girls (68 %vs32 %). There was no significant difference in gender between two groups. In 18 cases (56 %) hemangioma was present in the head and neck, 10 cases (31%) in the trunk and 4 cases (12.5%) in the extremities. No patient had multiple hemangioma. Patients had no previous medical treatments for hemangioma.

**Table 1:** Baseline characteristics of patients

value	Characteristic
**39.6 ± 4.4** **19** **10** **3** **68** **) ** **22)** **7.9** **±111**	Age (months) 0-5 yr 5-10 yr 10-15 yr Female SBP (mmHg)
**62± 6.2**	DBP (mmHg)
**27 ± 4.2**	Respiratory rate (breaths/min)
**115 ± 1.3** **18 (56)** **10 (31)** **4 (12.5)**	Heart rate (beats/min) Head and neck hemangioma Trunk hemangioma Extremitie shemangioma

Data are shown in mean ± SD or number (%)

**Figure 2:**
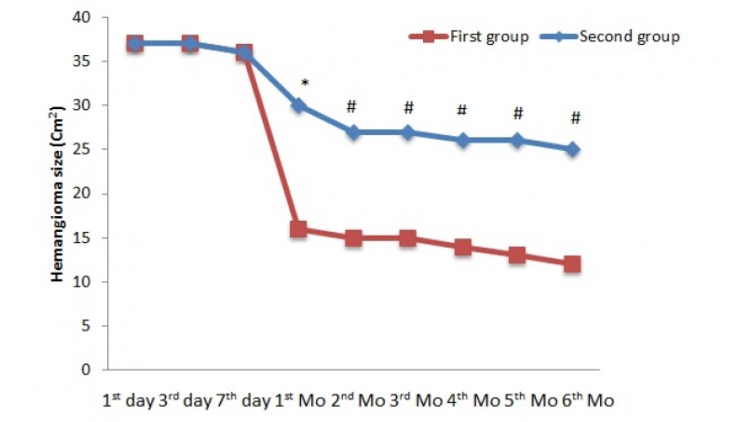
Changes in hemangioma size (Cm^2^) in the first phase of treatment; ^*^ P <0.01, ^#^ P <0.05

**Figure 3:**
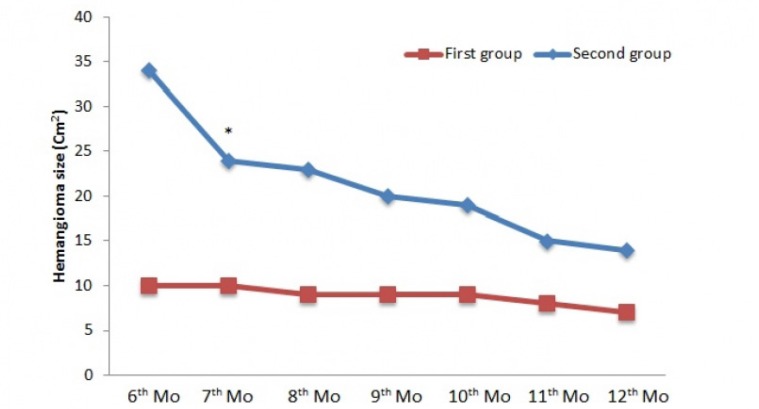
Changes in hemangioma size (Cm^2^) in the second phase of treatment; ^*^ P < 0.05.

*Efficacy*

In the first phase, at baseline, there was no significant difference in hemangioma size between observation and treatment groups. As shown in Figure 2, after 1 month of treatment, a significantdecrease was present in hemangioma size (30 cm^2^vs 16 cm^2^, *p *< 0.01). At other intervals,significant differences were present between observation and treatment groups (*p *< 0.05). 

In the second phase, as shown in Figure 3, a significant difference was seen in the 7thmonth of treatment between two groups (*p*< 0.05). In spite of decrease in hemangioma size, no significant difference was seen in other intervals. In the 1st month of treatment, a significant difference was noted in range of age; children who were in 0 to 5 years of age had quicker response compared to children who were in 5 to 15 years of age (14.3 ± 3.7 vs 26.4 ± 1.3 (cm^2^), *p *< 0.01). Seven (22 %) patients did not respond to the treatment. No case of regrowth was seen during treatment and within a-6 month follow-up.

*Safety*

None of the cases in two groups had any side effects related to propranolol therapy during one year of treatment and observation. 

## Discussion

To the best our knowledge, this is the first randomized, and cross over trial on propranolol effects for treating hemangioma in children up to 15 years old. Our findings suggest that oral dose of propranolol, 2 mg/Kg, is associated with decrease in size of hemangiomas. At this dose, no case of regrowth was seen during treatment and within 6 months of follow-up. In addition, no adverse effect of propranolol therapy was observed. Our results correspond to previously published data and confirm in particular the efficacy of propranolol. Notably, we have studied children with age of 1 month to 15 years. Due to lack of high-quality randomized and controlled studies, current recommendations are not evidence-based. In the study by Goswamy, propranolol was effective during the first months of treatment but a reproliferation was seen during the drug therapy (10).Schiestl showed in his work that 10 months use of oral propranolol was effective to reduce size of hemangioma in children aged from 1.5 to 9.1 months. In contrast to our work, 2 regrowth cases were reported during treatment (11). A work by Labreze showed that 6 months treatment with 3 mg propranolol oral solution is effective in infants 1 to 5 months of age. He reported propranolol related side effects like bradycardia and hypotension. In addition, 11% of the study subjects needed additional treatments (12). A similar investigation with smaller sample size by Hogeling proved efficacy of propranolol solution in children with ages from 9 weeks to 5 years. A few cases of bronchiolitis and respiratory infections were reported in his work (13). Furthermore, the study by Bertrand demonstrated efficacy of propranolol versus prednisone in children with 3.5 months of age. It is of note that the sample size was too small. The study showed that propranolol was associated with good or excellent response compared to prednisone which was associated with mild to moderate response (14).Compared to those investigations, different age range was used in our study. In the current work, children who were younger than 5 years had quicker response than patients who were between 5 to 15 years. This difference has not been reported elsewhere. It is not known with certainty that how propranolol could affect young children more quickly. In our study, 7 (22%) children did not respond to the treatment; however, some reports have shown full response to propranolol therapy. In the present study, no hemangioma regrowth was observed during six months follow-up. In the studies by Chik and Xiao, relative hemangioma recurrence was observed in several cases, which required further treatments (15, 16). It is noteworthy that none of our patients showed any serious complications related to propranolol during2 phases of the study. Side effects such as hypotension, bradycardia, shortness of breath, and lethargy have been reported in several investigations (17-19).Mechanism of action of propranolol in treatment of hemangiom has been extensively investigated. In the study by Hadashick, it has been shown that infantile hemangiomas have a large amount of beta 2 receptors (20). Support for this finding, comes from Wong’s work which revealed presence of both beta 1 and beta 2 receptors in hemangiomas (21). Propranolol blocks beta 1 and beta 2 and reduces the blood supply of the lesions. This effect is responsible for discoloration and softening of hemangiomas. In addition, inhibition of angiogensis and induction of apoptosis are other proposed mechanisms of action of propranolol for treatment of hemangioma. Angiotensin II has a pivotal role in hemangioma. It can upregulate factors that are necessary for proliferation and angiogenesis of the lesions; on the other hand, it can up regulate anti apopototic factors (22). Propranolol ability to suppress levels of rennin, may partially explain other modes of actions. In addition, propranolol can reduce levels of matrix metalloproteinase-9which is an important enzyme for angiogenesis (23). The exact mechanism of action of propranolol in hemangioma remains to be properly addressed.

## Conclusion

This trial suggests that propranolol at dose of 2 mg/Kg/day is beneficial in treating pediatric hemangioma with an acceptable safety profile.

## Study limitations

The present study has a number of limitations that should be acknowledged. As this was a single-arm study, further investigations with a randomized control study are necessary. In addition, due to a small number of patients in our study, efficacy and safety of propranolol should be tested in large and high-quality randomized control trials.

## Conflict of Interest

None.
